# Development of low-cost non-contact structural health monitoring system for rotating machinery

**DOI:** 10.1098/rsos.172430

**Published:** 2018-06-20

**Authors:** Vanraj, Robin Singh, S. S. Dhami, B. S. Pabla

**Affiliations:** 1 Department of Mechanical Engineering, National Institute of Technical Teachers Training and Research, Chandigarh, India; 2 Research & Development, ACOEM Group, ACOEM India, Vadodara, India; 3 Department of Electronics and Communication Engineering, Punjab Engineering College (Deemed to be University), Chandigarh, India

**Keywords:** non-contact structural health monitoring, acoustic signatures, microcontroller, data acquisition system, rotating machinery

## Abstract

Condition monitoring systems are increasingly being employed in industrial applications to improve the availability of equipment to increase the overall equipment efficiency. Condition monitoring of gearboxes, a key element of rotating machines, ensures to continuously reduce and eliminate costs, unscheduled downtime and unexpected breakdowns. This study demonstrates a low-cost microcontroller-based non-contact data acquisition system for condition monitoring of rotating machinery. Experimental validation of the proposed system was carried out by performing examination tests on a gearbox test rig. A user-friendly graphical user interface was also developed which facilitates users to perform signal processing in both real-time and offline mode. The proposed system can perform most of the functions available in complex, stand-alone vibration analysers. The use of a general-purpose PC and standard programing language makes the system simple, economical and adaptable to a variety of problems. The tests show the developed system can perform properly as proposed.

## 1. Introduction

The rapid development of engineering and mechanical components, together with the increase in complexity, cost of acquiring equipment and machine downtime, has attracted the attention of researchers towards the development of non-contact structural health monitoring (SHM) systems. Transformation of modern manufacturing technologies from mass production to lean manufacturing opens a new paradigm to implement the SHM for the improvement in safety, reliability, economy and intelligence against catastrophic failure applied in real-time environments [1,2].

Gears, a vital component of the transmission system, contributes to wide range of applications such as industrial, automotive and daily-life applications and productivity of the system is decreased with failure of any of the machine part. Catastrophic failures related to gears are a result of improper operating conditions and loading, thus leading to failure of the whole mechanism. According to the latest survey [3], the premature failure of gears and bearings has a large share of more than 95% in the breakdown of wind turbine gearboxes. Appropriate maintenance strategy can reduce the unplanned stoppages and minimize the number of failures, thereby reducing serious and costly consequences.

### 1.1. Acoustic as a diagnostic tool

Not only are the current conditions monitored by diagnosis tools, but future conditions of machines are also predicted while in operation. Therefore, the information must be collected externally without affecting the working of machine. Various monitoring approaches like direct and indirect techniques can be put on to any application by adaptations and alterations, to accomplish an acceptable level of consequences. Direct techniques, such as vision and optical methods, measure the actual geometric changes in the mechanical component [4]. These techniques are not used in real-time applications because the machine has to be stopped for physical inspection. Indirect techniques *viz.* vibration, oil analysis, thermography, laser vibrometer, acoustic analysis and motor current signature analysis are easy to implement. However, vibration, thermography and acoustic monitoring are generally practiced as they reflect the status of the machine instantaneously. Researchers have used measurements of vibrations [5–7,7–12], acoustics [5,13–22] and thermography [23–25] to estimate the gearbox condition. In contrast to the aforementioned variables, vibration is one of the features of modern mechanical machinery that is now continuously monitored in many significant applications. However, acoustic-based condition monitoring provides some technical advantages over vibration-based methods. Firstly, acoustic sensors can be placed at any convenient location on the periphery of the monitored system, whereas the vibration monitoring technique requires surface contact placement in specific orientation to get accurate and meaningful information. Secondly, acoustic monitoring is more sensitive to vibrating bodies than vibration sensors, and hence provides an opportunity to identify faults at an early stage [2,20–22,26,27]. Successful implementation of non-contact incipient fault diagnosis of fixed-axis gearboxes has been carried out [28–32].

### 1.2. Structural health monitoring

SHM is a procedure that aims to estimate the structure condition, fault detection and problem diagnostics in the machine by means of the evaluation of some measured physical features. Microphones are the most widely used non-contact-type instrument to measure the acoustic emission from mechanical components. An effective SHM system can improve productivity and ensure work-piece quality contributing to a major influence on machining economics. The effectiveness of the SHM system relies on the sensitivity of sensor used, position of sensor mounting, number of sensors to be used and types of sensors to be practiced. Current health monitoring approaches are much able to determine the [33]:

— presence of the fault in the mechanical component (level 1);
— geometric location of the fault (level 2);
— level/severity or type of fault (level 3); and
— anticipation of the remaining lifetime (level 4).

The present work develops a non-contact SHM system using a low-cost microcontroller-based data acquisition system. The system used the microphone to measure the acoustic signal of the gearbox. The produced acoustic signal is then analysed using signal analysis graphical user interface (GUI) developed in Matlab${\;}^{®}$ 2016a to determine the health of the machine. Experiments are carried out on a gearbox test-rig to collect data for the analysis. This study proposes to develop a cost-effective data acquisition system for monitoring the gearbox condition. The objectives of this work include the following:

(i) to develop an economical non-contact acoustic sensor-based device to monitor the current status of the gearbox for reliable SHM using microcontroller interfaced with Matlab;
(ii) to correlate the vibration signatures with machine health; and
(iii) to develop a GUI for easy implementation of SHM.

## 2. Proposed data acquisition system structure

**Figure 1.**
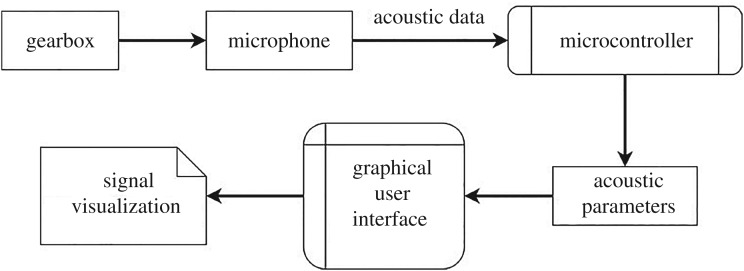
Information flow for non-contact acoustic measurement.

The functional information flow of the proposed data acquisition system is shown in figure 1.

## 3. Microcontroller-based structural health monitoring data acquisition system

Acoustic data acquired through the data acquisition system was analysed and implemented for gearbox condition monitoring. The hardware system development, software system development, integration and testing of the data acquisition system, and calibration along with the existing data acquisition system is described in the following sections.

### 3.1. Hardware system development

The non-contact data acquisition system employed off-the-shelf equipment in a novel integrated approach. During gearbox operation, real-time acoustic data were collected through a microphone. The raw signal was processed to extract the characteristic features from the gearbox signals. The various equipments that are used for developing a system are described below.

#### 3.1.1. Test rig

**Figure 2.**
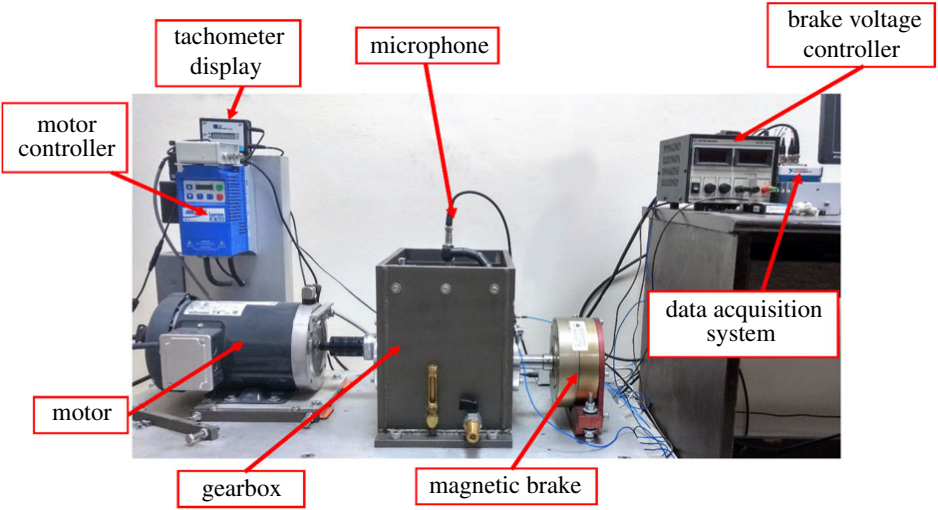
Test rig used for experimental measurements.

A test rig, gearbox diagnostics simulator was used for simulating faults in rotating machinery. The test rig had a feature to simulate industrial drive-trains, especially as an experimental research tool. Figure 2 shows the experimental set-up used for studying gear faults. The set-up has been designed to simulate real working conditions of a gearbox. The detailed specifications of the test rig are listed in table 1. Table 2 summarizes the specifications of the gearbox.

**Table 1.** Test rig specifications.

parameters	values/description
driving motor	3 HP, 3-phase
variable frequency drive	Lenze
speed measurement	tachometer
rotational speed	100 r.p.m. to 3600 r.p.m.
external loading	magnetic loading
loading capacity	0.126 N m to 24.85 N m
speed reduction	single stage
reduction ratio	3.44:1

**Table 2.** Gearbox specifications.

specification	values
length	27.5 cm
width	19 cm
height	26.5 cm
number of teeth on driver	29
number of teeth on driven	100
spur gear pressure angle (*α*_*p*_)	20^°^

#### 3.1.2. Acoustic sensor

For acoustic signal acquisition a general-purpose microphone (Ahuja GN45) was used. The microphone gave the output in sound pressure level, i.e. Pascals (Pa). The position of the microphone is fixed at 24 cm vertically from the bottom of the set-up near the meshing pair in accordance to the optimal sensor location experimentation conducted by Vanraj *et al.* [34,35]

#### 3.1.3. Microcontroller-based data acquisition device

**Figure 3.**
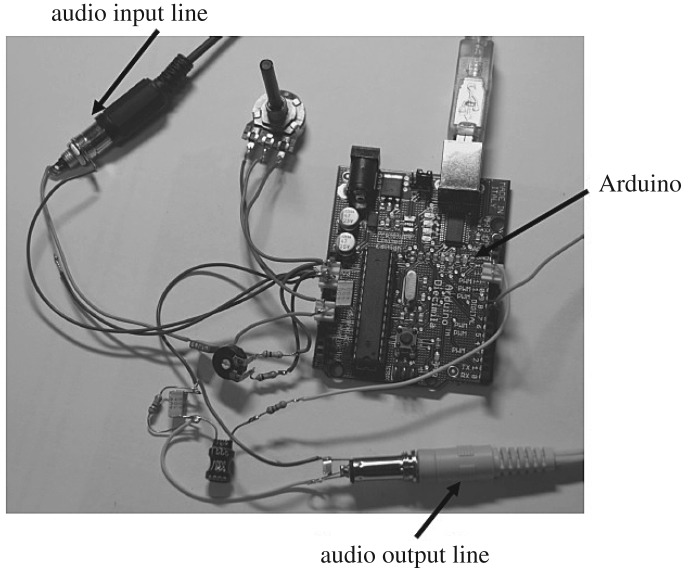
Schematic of the non-contact acoustic measurement circuit.

A microcontroller-based data acquisition device (Arduino) has been used to acquire data. This device is interfaced to computer program using Matlab via a USB port. The device enables acquisition of acoustic signals using a low-cost hardware by using a standard high-speed USB port and PC-based analogue and digital input/output data acquisition. A Matlab microcontroller is associated with the sensor, and brings forth a digital output after burning the program for the microphone. Figure 3 shows the schematic of the non-contact acoustic measurement circuit.

**Figure 4.**
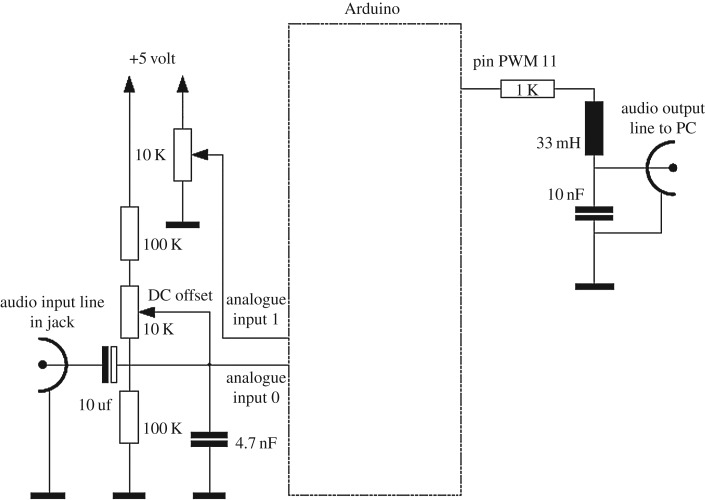
Circuit diagram of component integration.

Figure 4 shows the circuit diagram of various component integration. Arduino analogue Pin0 is used to collect the audio input signal. To control the audio effects, a potentiometer connected to analogue input 0 was used. Pin 11 on microcontroller board was used as pulse-width-modulation (PWM) audio output for recording the audio signal in Matlab.

### 3.2. Graphical user interface development

**Figure 5.**
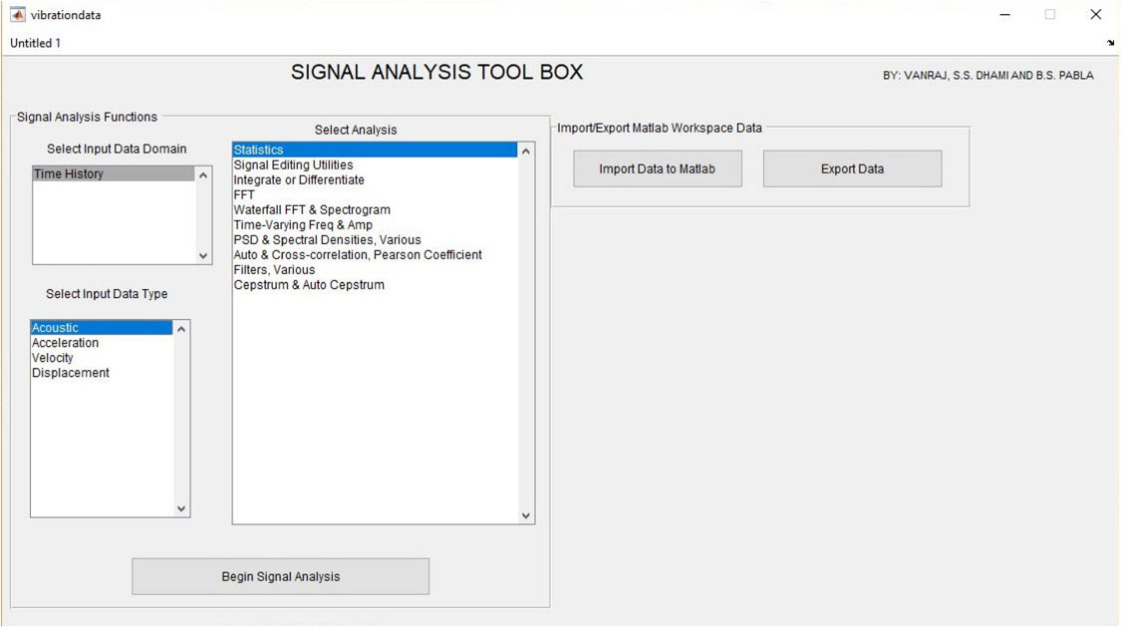
Graphical user interface developed for acoustic signal analysis.

A Matlab code was developed which facilitates acoustic data acquisition, online or offline signal processing, and graphical display of the recorded signals. Because the range of frequencies of interest available in acoustic signals are generally of the order of several kilo-Hertz, thus it is compulsory to keep the data acquisition and analysis cycle time very short. This has been achieved with the help of fast executing algorithms and computer programs in Matlab. The developed GUI was successfully interfaced with the hardware system for real-time data acquisition. Figure 5 shows the GUI developed for acoustic signal analysis. The developed GUI facilitates the user to compute frequency spectra, cepstrum analysis, signal editing of vibration, acoustic and velocity, archiving data in various ASCII formats. Both real-time signal processing and offline signal processing can be carried out for SHM of machinery.

### 3.3. Simulated faults

**Figure 6.**
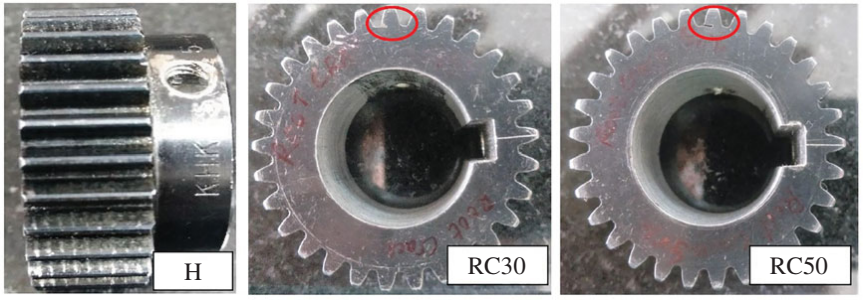
Gears with different tooth removal.

Since the time period to overhaul a new gearbox may vary from six months to 1 year depending upon working conditions, naturally generated faults and their detection becomes difficult. Therefore, the only option left is to study seeded fault trials in gearboxes. Most common gearbox faults can be categorized as: (i) root crack, (ii) surface spalling, and (iii) chipped tooth. The chipped tooth is the rupture of material from the working tip of a gear. Root crack is extremely common in numerous industrial practices which triggers other gear faults and is very difficult to detect at its initial stage [27]. Spur gears with simulated root crack damage generated by wire electrical discharge machining in steps based on per cent cutting of teeth root have been considered in the present study. Three types of gear conditions are investigated, *viz.* a healthy gear, a gear with 30% root crack and a gear with 50% root crack as shown in figure 6. The faults were induced on the pinion mounted on the input shaft. The notation of all running condition is listed in table 3. The noise condition of the test laboratory was ambient office working conditions with general machines such as fans, air conditioner, etc., switched on during experimentation. To achieve repeatability of measurement, each experiment was repeated five times and then the averaged value was used in the final results. A total of 30 k data points were collected for each gear state at running speed of 15 Hz, 25 Hz and 35 Hz.

**Table 3.** Fault description.

fault description	notation
healthy	H
root crack 30%	RC30
root crack 50%	RC50

### 3.4. Calibration

In order to calibrate the results obtained using the developed system, a NI-cDAQ-9178 data acquisition system was used to acquire the acoustic signals using a G.R.A.S 46AE microphone under the same gearbox operating conditions. The sampling frequency of the NI-DAQ was 12.8 kHz and 30 k data points were collected for each case. Time-domain and frequency-domain-based comparison was done to check the accuracy of the proposed system. Mostly used statistical parameters [36] were extracted from the signals for each gear running condition.

## 4. Results and discussion

Based on the analysis and the theoretical framework provided in the earlier sections, the experiment has been performed on the test rig, and results obtained from the analysis phase in the development of a non-contact SHM system for gearboxes are discussed in this section. A number of experiments have been conducted to check the accuracy in the measurement of the developed system by calibrating it with a standard microphone and data acquisition system.

### 4.1. Time domain analysis

Firstly, the time domain acoustic signals for different gear conditions recorded using a microcontroller-based data acquisition device and NI-cDAQ-9178 were compared. Three statistical parameters, *viz.* root mean square (RMS), kurtosis and crest factor were calculated for each gear condition. Annotations used for graphical representation are as follows:

— microcontroller-based data acquisition device using general-purpose microphone: -GN45; and
— NI-cDAQ-9178 using a G.R.A.S microphone: -46AE

**Figure 7.**
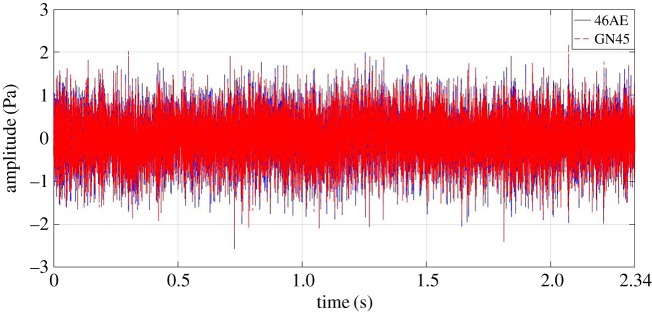
Comparison of acoustic signal collected from a healthy gear using a GN45 microphone and a 46AE microphone at 35 Hz rotational speed.

For demonstration purposes, 35 Hz rotational speed time domain data are highlighted. Figure 7 shows the comparison of variation in acoustic amplitude for a healthy gear measured by both 46AE and GN45 acoustic sensors at 35 Hz rotational speed. It has been observed that the developed system gives almost the same values like the standard data acquisition system. Following points were observed for healthy gear condition:

— for 46AE sensor, the maximum and minimum values were 1.772 Pa and −2.303 *Pa*, respectively; whereas, for GN45 sensor, the maximum and minimum values were 1.937 Pa and −2.162 *Pa*, respectively; and
— RMS value of acoustic data obtained from 46AE and GN45 were 0.4789 and 0.4791, respectively.

**Figure 8.**
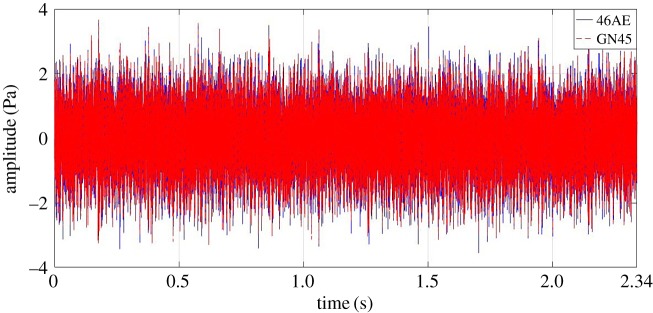
Comparison of acoustic signal collected from RC30 using a GN45 microphone and a 46AE microphone at 35 Hz rotational speed.

Figure 8 depicts the comparison of variation in acoustic amplitude for RC30 measured by both 46AE and GN45 acoustic sensors. Following points were observed for the RC30 gear condition:

— for 46AE sensor, the maximum and minimum values were 3.644 Pa and −3.555 *Pa*, respectively; whereas, for GN45 sensor, the maximum and minimum values were 3.664 Pa and −3.304 *Pa*, respectively; and
— RMS value of acoustic data obtained from 46AE and GN45 were 1.028 and 1.021, respectively.

**Figure 9.**
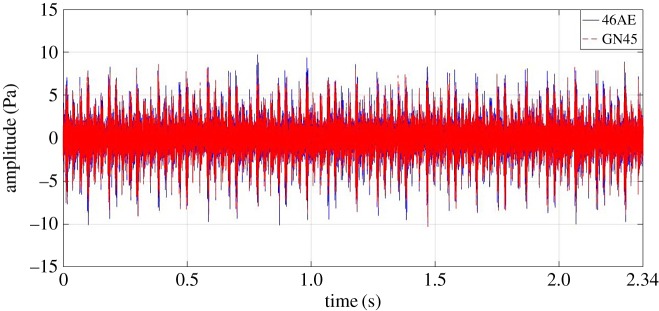
Comparison of acoustic signal collected from RC50 using a GN45 microphone and a 46AE microphone at 35 Hz rotational speed.

Figure 9 depicts the comparison of variation in acoustic amplitude for RC50 measured by both 46AE and GN45 acoustic sensors. Following points were observed for the RC50 gear condition:

— for 46AE sensor, the maximum and minimum values were 9.743 Pa and −10.33 *Pa*, respectively; whereas, for GN45 sensor, the maximum and minimum values were 8.942 Pa and −10.3 *Pa*, respectively; and
— RMS value of acoustic data obtained from 46AE and GN45 were 2.09 and 2.001, respectively.

**Figure 10.**
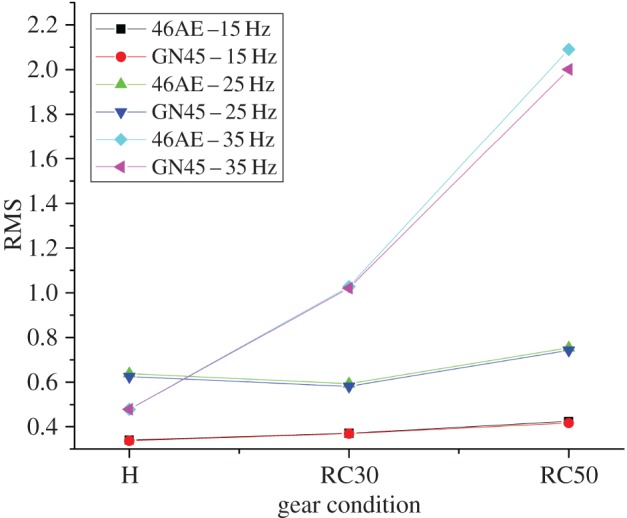
Variation of RMS for different gear conditions at various speeds.

**Figure 11.**
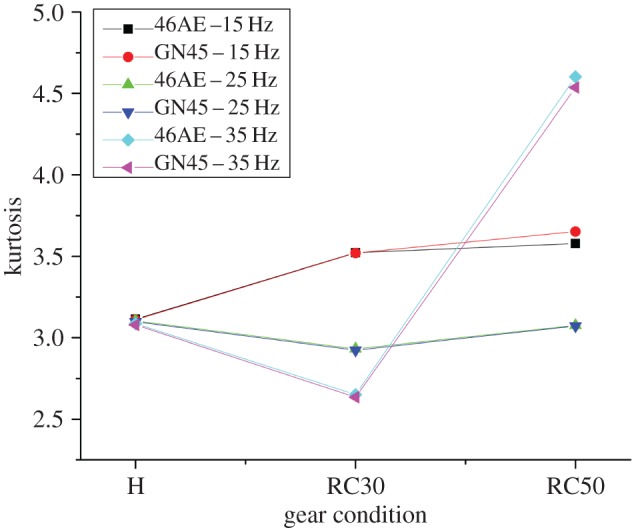
Variation of kurtosis for different gear conditions at various speeds.

**Figure 12.**
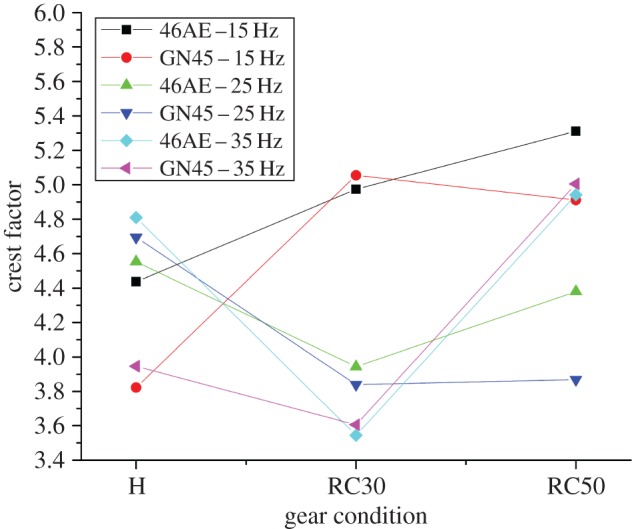
Variation of crest factor for different gear conditions at various speeds.

The variation of calculated statistical parameters for different gear conditions at various speeds is shown in figures 10–12.

It was observed that the statistical parameters are well separated for different rotational speeds for all gear conditions. Also, the values of statistical parameters for both the acoustic sensors were found to be close to each other for each gear condition at all rotational speeds. It implies that the proposed system could acquire the acoustic data with the same accuracy as that of existing analysers. The per cent deviation of statistical parameters obtained using two acoustic sensors is listed in table 4.

**Table 4.** Percent deviation of statistical parameters at different gear conditions and rotational speeds for both acoustic sensors.

parameters	gear condition	46AE-15Hz	GN45-15Hz	per cent deviation	46AE-25Hz	GN45-25Hz	per cent deviation	46AE-35Hz	GN45-35Hz	per cent deviation
RMS	H	0.339	0.336	0.854	0.638	0.624	2.069	0.478	0.479	−0.042
	RC30	0.370	0.368	0.405	0.593	0.581	1.956	1.028	1.021	0.681
	RC50	0.424	0.416	1.862	0.753	0.742	1.460	2.09	2.001	4.258
kurtosis	H	3.115	3.11	0.161	3.104	3.098	0.193	3.087	3.079	0.259
	RC30	3.522	3.52	0.057	2.932	2.924	0.273	2.651	2.635	0.604
	RC50	3.578	3.652	−2.068	3.076	3.072	0.130	4.602	4.536	1.434
crest factor	H	4.437	3.823	13.838	4.553	4.695	−3.119	4.81	3.947	17.942
	RC30	4.974	5.055	−1.628	3.944	3.84	2.637	3.544	3.606	−1.749
	RC50	5.311	4.911	7.532	4.38	3.869	11.667	4.941	5.005	−1.295

It was observed that the per cent deviation for RMS and kurtosis is under acceptable range (under 10%) for all running conditions, however, per cent deviation for crest factor was little high (above 10%).

### 4.2. Frequency domain analysis

**Figure 13.**
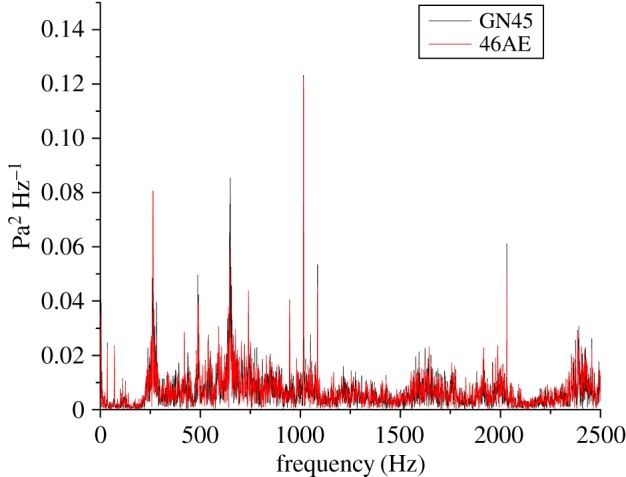
FFT of healthy gear for a GN45 microphone and a 46AE microphone.

**Figure 14.**
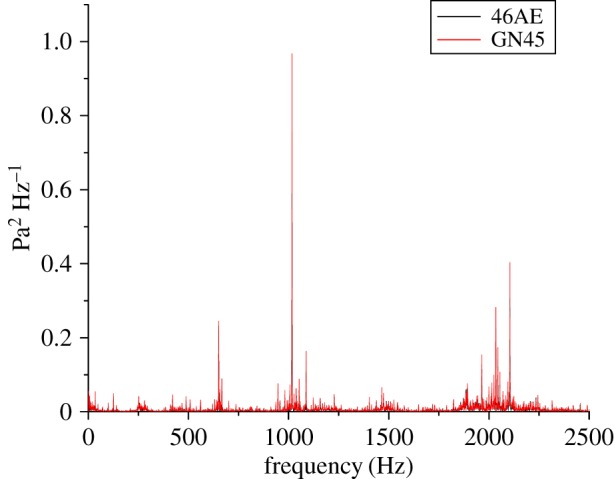
FFT of RC30 for a GN45 microphone and a 46AE microphone.

**Figure 15.**
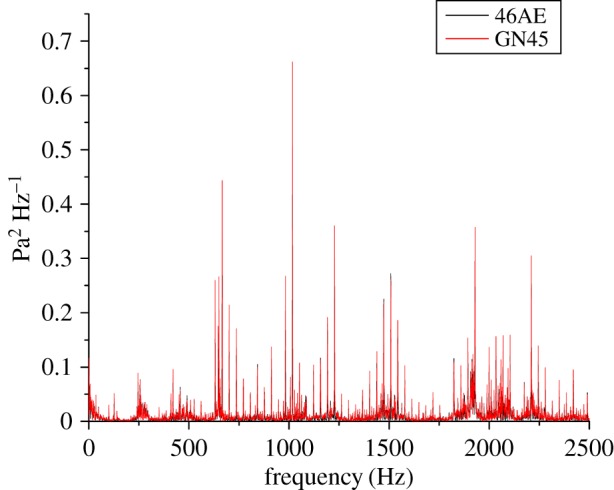
FFT of RC50 for a GN45 microphone and a 46AE microphone.

Time-domain analysis, because of its ease in implementation, had been used in most of the past research for analysis of the machining signals. However, frequency domain analysis facilitates in isolating a specific frequency component which is related to a specific machine component or fault. For this work, the fast Fourier transform (FFT) was applied to extract the features from the acoustic data as shown in figures 13–15. Gear mesh frequency (GMF) is generally visible even for a healthy gear in the FFT spectrum when gears are rotated at a particular speed. However, the amplitude of GMF and its harmonics are influenced by the presence of a fault on the meshing gear teeth. The theoretical values of GMF may vary from actually obtained values owing to speed fluctuations [27,37,38]. For example, theoretical GMF and its harmonics calculated at 35 Hz of input shaft are:

4.1 GMF=number of teeth on pinion×rotational frequency of pinion (Hz).

Therefore,

$$\begin{array}{r} \text{GMF (first harmonic)}=29\times 35=1015\;\mathrm{Hz}\\ \text{2 GMF (second harmonic)}=2\times 1015=2030\;\mathrm{Hz}. \end{array}$$

The peak values of FFT spectrum at different frequencies for different gear conditions running at 35 Hz rotational speed obtained for both acoustic sensors, i.e. 46AE and GN45 has been tabulated in table 5.

**Table 5.** FFT spectrum amplitude for 46AE and GN45 acoustic sensors at 35 Hz rotational speed.

gear condition	frequency of peak amplitude (46AE) (Hz)	FFT spectrum peak amplitude (*Pa*^2^) *Hz*^−1^	frequency of peak amplitude (GN45)(Hz)	FFT spectrum peak amplitude (*Pa*^2^) *Hz*^−1^
H	1015	0.122	1015	0.123
RC30	1017	0.794	1017	0.967
RC50	1017	0.542	1017	0.661

It was observed that the peak amplitude of FFT spectrum in all three cases, i.e. H, RC30 and RC50 for both the acoustic sensors lie around 1015 Hz. A close agreement was observed of the frequency spectra obtained from the two acoustic sensors. Similar results were obtained for other rotational speeds, i.e. 15 Hz and 25 Hz.

## 5. Features evaluation and economics analysis

Features evaluation and economics analysis of the proposed SHM system was conducted by comparing the features with state-of-the-art benchtop equipment available in the market. Both hardware and software capabilities were compared as listed in table 6. The proposed system has a variety of software features incorporated using Matlab algorithms, such as signal editing utility, FFT, waterfall and spectrogram, power spectral density, cepstrum and graphic display of data. With these features the proposed system can perform most of the functions available in complex, stand-alone analysis devices.

**Table 6.** Features comparison of various vibration analysers.

	features	proposed system	NI-cDAQ-9178 [39]	Oros OR34 [40]	Pröftechnik Vibguard [41]
hardware	no. of channels	depend upon no. of hardware devices	depend upon no. of hardware devices	4	16
	portable	✓	✓	✓	✓
	system weight	approx. 500 g	1 kg	1.4 kg	2 kg
	PC requirement for signal analysis	✓	✓	✓	✓
	all true channels	χ	χ	✓	✓
software	open source	✓	χ	χ	χ
	module for acoustic analysis	✓	✓	✓	χ
	flexibility for addition of new signal analysis modules	✓	χ	χ	χ
	offline signal analysis	✓	✓	✓	✓
approximate cost		$500	$8000	$33 000	$45 000

## 6. Conclusion

A microcontroller data acquisition device based on non-contact transducer has been developed for fault detection and preventive maintenance of rotating machinery. The present work has aimed to develop a general-purpose SHM device which is simple, economical and adaptable to individual problems. The system can perform most of the functions available in complex, stand-alone vibration analysers. The software is menu-driven and user-friendly. The device is capable of computing frequency spectra, cepstrum analysis, signal editing of vibration, acoustic and velocity. The proposed SHM device has been used for fault detection in gearboxes. The following conclusions have been obtained from the experimentation and analysis of results:

(i) real-time non-contact condition monitoring of rotating machinery can be carried out by using the developed system;
(ii) owing to non-contact data acquisition, the drawback of traditional sensors that have to be mounted on the machine has been diminished;
(iii) the results were also compared and calibrated with the standard data acquisition device and acoustic sensor for the validation of results. It has been found that the developed system showed the same pattern of variation of acoustic amplitude as an standard system does;
(iv) the FFT spectrum peak values for different gearbox running conditions were observed. It has been noticed that peak amplitude of FFT spectrum in all three cases, i.e. healthy, RC30 and RC50 for both the data acquisition system lie near the GMF; and
(v) economic analysis revealed that the developed data acquisition system is economical, user-friendly and equipped with various signal processing tools required for health monitoring of rotating machinery.

## 7. Research trends

(i) Future research may focus on integrating artificial intelligence as an integral part of SHM systems with the use of emerging Information Communication Technologies to give real-time decision regarding status of the machine.
(ii) The developed system can be used to determine the machine health for heat transport system components of a nuclear reactor, aircrafts, turbines, power plants, etc.
(iii) Authors also suggest robustness evaluation of the proposed system by implementing it in real-world applications.

## Data accessibility

The original data used in this article can be assessed from the Dryad Digital Repository (https://doi.org/10.5061/dryad.n0d07ds) [42].
